# Controlled release fertiliser as an alternative nutrient management strategy for immature oil palm establishment on steep terrain: growth and foliar nutrient responses

**DOI:** 10.3389/fpls.2026.1846207

**Published:** 2026-06-15

**Authors:** Bernard Huang Chai Khoo, Yu Yang Chang, Choon Cheak Sim, Siti Aishah Abd Wahid, Mohamad Hazim Nazli, Mohd Firdaus Mohd Anuar

**Affiliations:** 1 Department of Plantation Research and Advisory, SD Guthrie Research Sdn Bhd, Pulau Carey, Selangor, Malaysia; 2 Faculty of Agriculture Science, Department of Plant Science, Universiti Putra Malaysia, Serdang, Selangor, Malaysia

**Keywords:** controlled release fertiliser (CRF), early-stage nutrient management, nutrient optimisation, oil palm, sloping agriculture land

## Abstract

**Introduction:**

Controlled-release fertilisers (CRFs) are increasingly considered a viable alternative to conventional fertiliser management in oil palm plantations, particularly in topographically challenging environments. However, their efficacy during the early establishment phase under steep terrain conditions remains insufficiently documented.

**Methods:**

A field trial was conducted using a randomised complete block design (RCBD) to evaluate the effects of different CRF rates and fertiliser regimes on immature oil palms over the first 12 months after planting (MAP). CRF was applied as a single annual basal dressing at 500 g, 700 g, and 1,000 g palm^-1^, and compared against conventional split applications of 5.0 kg palm^-1^ compound fertiliser. Vegetative growth parameters and foliar nutrient concentrations were assessed at 6 and 12 MAP.

**Results:**

Vegetative growth parameters were generally not significantly different among treatments, except for rachis length, which varied significantly (p < 0.05). Foliar nutrient concentrations showed greater response, with significant treatment effects observed for N, P, K, and Mg at 6 MAP, and N, P, and K at 12 MAP, while foliar B and rachis K were not significantly affected. CRF at 500 g palm^-1^ maintained nutrient levels within acceptable ranges and supported comparable growth.

**Discussion:**

These findings indicate that single-application CRF can sustain early oil palm establishment under hilly terrain conditions, providing comparable performance to conventional fertilisation during the first year after planting.

## 1 Introduction

Oil palm (*Elaeis guineensis* Jacq) is one of the world’s most economically significant perennial crops, supplying more than one-third of global vegetable oils and sustaining diverse food and non-food industries due to its oxidative stability and versatile chemical properties (Singh et al., 2022). Major importing region including China, Southeast Asia, and parts of Europe continue to rely heavily on palm oil as a staple commodity (Rival and Levang, 2015). The crop’s exceptionally high land-to-oil productivity, derived from both the mesocarp and kernel fractions, and its long commercial life cycle of approximately twenty-five years, position oil palm as a critical resource in global food security (Murphy et al., 2021). Modern commercial plantings are predominantly the Dura × Pisifera (DxP) hybrid, producing the high-yielding Tenera type characterized by a thinner shell and superior oil extraction efficiency (Singh et al., 2022).

Beyond traditional edible oil markets, oil palm is increasingly recognized for its potential contribution to renewable energy pathways, including biodiesel and biogas production (Rival and Levang, 2015).Yet expansion-driven cultivation presents well-documented ecological trade-offs. In regions where new plantations replace primary forests, significant biodiversity loss, hydrological disruption, and elevated greenhouse gas emissions have been reported, underscoring the challenge of aligning productivity with sustainability (Rahman, 2022; Herdiansyah et al., 2024). Consequently, contemporary agronomic discourse has shifted toward intensification on existing cultivated land rather than geographical expansion (Rival and Levang, 2015).

This shift is particularly relevant in Malaysia and Indonesia, where oil palm is widely established on highly weathered Oxisols and Ultisols soils that are acidic, low in fertility, and prone to aluminium toxicity, nutrient imbalance, and leaching (Shamshuddin and Daud, 2011; Sham et al., 2024). These edaphic constraints weaken root development and reduce nutrient-use efficiency, creating substantial fertiliser management challenges even in well-managed estates. While early growth requires moderate nutrient inputs, the subsequent bunch development stage demands continuous supplementation of nitrogen, phosphorus, potassium, magnesium, boron, zinc, and copper to support biomass accumulation and maximize yields during the critical peak production window between 7–15 years old (Fairhurst and Härdter, 2003a; Goh et al., 2003).

Conventional fertiliser management in oil palm cultivation relies predominantly on repeated applications of inorganic fertilisers to compensate for the substantial nutrient losses caused by intense tropical rainfall and subsequent leaching (Sham et al., 2024). To enhance nutrient retention and support long-term soil fertility, these inputs are complemented by sustainable field management practices. Strategies such as systematic frond stacking, the application of empty fruit bunches (EFB), and the maintenance of leguminous cover crops contribute to the accumulation of soil organic carbon, which in turn improves soil structure, enhances cation exchange capacity (CEC), and promotes greater nutrient buffering and cycling within the soil-plant system. While these practices strengthen overall soil quality, they do not fully address fertiliser use inefficiencies associated with the rapid release of nutrients and their temporal mismatch with plant uptake dynamics.

These inefficiencies are particularly pronounced during the establishment phase of newly replanted palms during which early vegetative growth and root system expansion determine the physiological foundation for productivity across the subsequent plantation lifecycle. This challenge is further intensified in hilly or steep terrain, where repeated fertiliser applications are operationally demanding, costly, and prone to increased nutrient losses through runoff. Steep slopes promote rapid surface water flow and reduced infiltration, accelerating the downslope movement of applied nutrients and resulting in uneven nutrient distribution within the field. Consequently, spatial variability in soil fertility may arise, with nutrient depletion in upper slope positions and accumulation in lower areas. In addition, limited accessibility reduces the consistency and precision of fertiliser application, further exacerbating nutrient management inefficiencies under such conditions (Afandi et al., 2017; Yosephine et al., 2025).

Controlled-release fertilisers (CRFs) have emerged as a promising alternative to conventional fertiliser regimes in perennial cropping systems. CRFs utilise semi-permeable polymer or resin coatings that regulate nutrient diffusion through osmotic processes, providing a gradual and sustained nutrient supply that can be synchronised with crop demand. Release longevity varies from weeks to approximately 12 months depending on coating composition and thickness (Azeem et al., 2014; Lawrencia et al., 2021). By moderating the rate of nutrient delivery, CRFs can mitigate leaching losses under high-rainfall tropical conditions, support early frond number and bole diameter, and enhance overall nutrient-use efficiency (Chien et al., 2009). In the context of oil palm replanting systems, CRFs offer a dual operational advantage: improved nutrient-use efficiency and a significantly reduced frequency of field applications, which is a particularly meaningful benefit in hilly replanting areas where labour and access constraints are greatest (Totok et al., 2024).

Despite these potential benefits, agronomic evidence for CRF performance in immature oil palms under field conditions, particularly across varied application rates and on sloped terrain remains limited. Therefore, this study aimed to evaluate the effects of different CRF rates and application regimes on the vegetative growth and foliar nutrient status of immature oil palms during the first 12 months after planting under steep terrain conditions, with the objective of determining whether CRF can serve as a practical and effective alternative to conventional fertiliser programmes during the early establishment phase.

## 2 Methodology

### 2.1 Experimental design and study site

#### 2.1.1 Study site

The study was conducted in an immature oil palm field in northern Peninsular Malaysia (4.8516°N, 101.0943°E). The site is characterized by undulating to hilly terrain and soil classified as Typic Paleudult with a heavy clay texture. Oil palms were planted in a triangular layout at a density of 136 stands per hectare. The study area records a mean annual precipitation of 2,106 mm. Baseline soil samples were collected prior to treatment application from two depths (0–15 cm and 15–30 cm). Soil pH, organic carbon (C), total nitrogen (N), total phosphorus (P), available phosphorus (P), exchangeable potassium (K), calcium (Ca), magnesium (Mg), and cation exchange capacity (CEC) were determined using standard analytical procedures. Extreme values were winsorized within each depth layer using a 3×IQR (Tukey) rule to minimize the influence of outliers. The initial soil properties are presented in **Table 1**.

**Table 1:** Initial soil chemical properties of the study site prior treatment application (mean ± SD).

Parameter	0–15 cm (n=42)	15–30 cm (n=42)
pH (H_2_O)	4.5 ± 0.2	4.4 ± 0.2
Organic C (%)	0.62 ± 0.14	0.63 ± 0.15
Total N (%)	0.084 ± 0.013	0.085 ± 0.012
Total P (mg kg⁻¹)	724.4 ± 248.0	692.2 ± 179.1
Available P (mg kg⁻¹)	42.9 ± 46.4	42.1 ± 49.3
Exchangeable K (cmolc kg⁻¹)	0.27 ± 0.12	0.27 ± 0.11
Exchangeable Ca (cmolc kg⁻¹)	0.67 ± 0.40	0.66 ± 0.34
Exchangeable Mg (cmolc kg⁻¹)	0.17 ± 0.10	0.17 ± 0.08
CEC (cmolc kg⁻¹)	4.66 ± 1.08	4.70 ± 1.02

1 Values are mean ± standard deviation (SD). Extreme values were winsorized within each depth layer using a 3×IQR (Tukey) rule.

#### 2.1.2 Experimental design and plot layout

**Figure 1:**
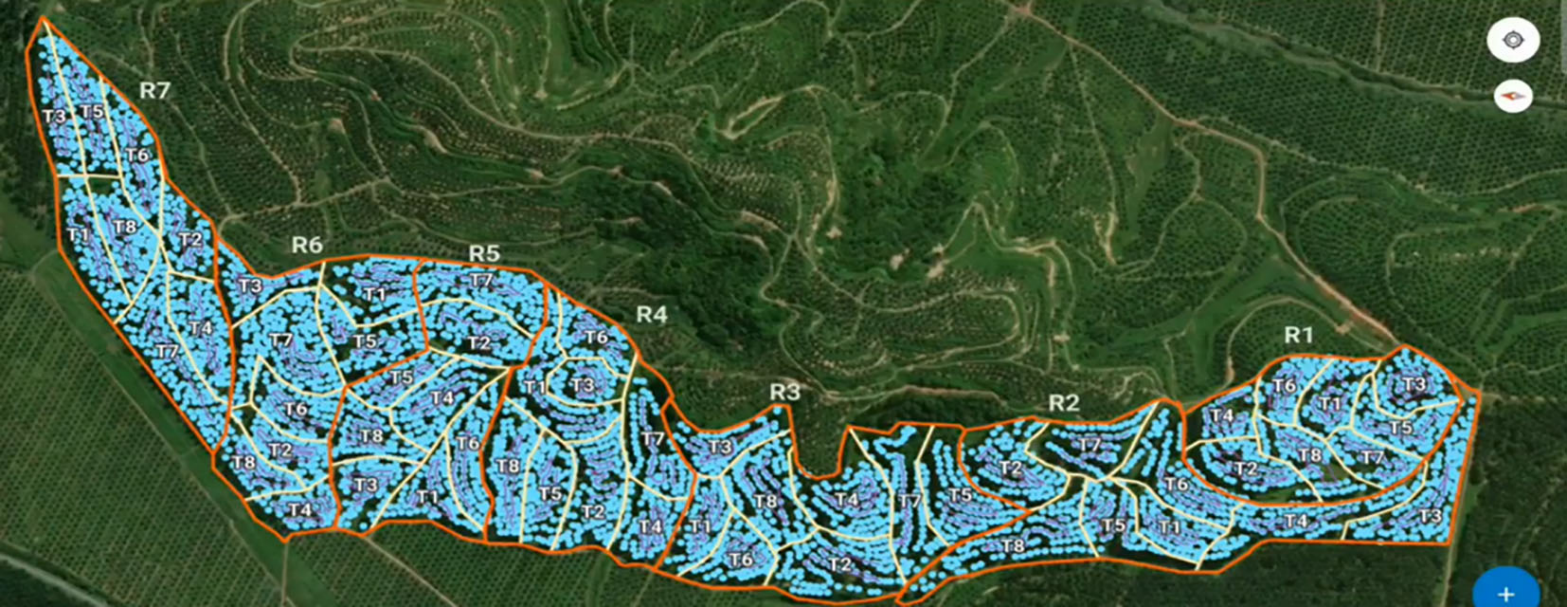
Location aerial view of trial location. An aerial map showing a large land area divided into seven regions labeled R1 to R7, representing replications. Each region is further segmented into treatment plots labeled T1 to T8. Blue dots densely fill the interior sections and represent individual oil palm trees. Thick orange lines outline the primary boundary of the study area, while yellow lines separate subplots within each region. The mapped zones are surrounded by dense green vegetation, indicating natural or uncultivated areas. The figure illustrates the experimental layout and spatial arrangement of replications and treatments within the plantation.

A Randomized Complete Block Design (RCBD) was employed to minimize the influence of topographical gradients across the experimental site (**Figure 1**). The site was divided into 56 plots, consisting of 8 treatments and 7 replications (**Table 2**). Within each plot, 15 central palms were designated as recording palms to minimize edge effects, thereby ensuring that the data collected represented the true treatment effects rather than variation caused by boundary influences. Altogether, 840 recording palms were used in the experiment.

**Table 2:** Treatment list.

Treatment	Fertiliser	Rate
1	CRF A	500 g
2	CRF B	500 g
3	CRF A	700 g
4	CRF B	700 g
5	CRF A	1000 g
6	CRF B	1000 g
7	Control- Compound fertiliser	5000 g
8	Mulching + Compound fertiliser	5000 g

### 2.2 Fertiliser/treatment applications

Controlled-release fertiliser (CRF) incorporated with 500 g Jordan rock phosphate (28% P_2_O_5_) was evaluated across six treatments, comprising two CRF formulations (CRF A and CRF B) applied at three rates: high (1.0 kg palm⁻¹), medium (700 g palm⁻¹), and low (500 g palm⁻¹). CRF A had a nutrient composition of 17:8:9:3MgO, while CRF B comprised 17:7:9:2MgO. Both formulations utilised biodegradable polymer coatings to regulate nutrient release under field conditions. All CRF treatments were applied once at planting, by placing the fertiliser mixture directly in the planting hole prior to transplanting. Treatment 7 served as the conventional fertiliser control and received 500 g rock phosphate at planting, followed by four split applications totalling 5 kg palm⁻¹ year⁻¹ of compound (12:12:17:2MgO) during the planting year. Treatment 8 evaluated fertiliser placement under mulch, where 5 kg compound fertiliser was applied beneath biodegradable cotton-based mulch sheets covering the palm circle after planting. The mulch measured approximately 1.5 m × 1.5 m per palm, with a mean thickness of 0.20 mm, and was applied to enhance soil moisture conservation and reduce surface evaporation.

### 2.3 Data collection

#### 2.3.1 Vegetative measurements

Vegetative measurements were recorded at 6 and 12 months after planting (MAP). The parameters measured included total frond number (TF, n), rachis length (RL), leaf area index (LAI), and petiole cross-section (PCS, cm²). A representative frond was selected to assess palm growth according to palm age. In this study, frond 9 was consistently selected at both sampling times. Rachis length (m) was measured from the base of the rudimentary leaflets to the tip of the rachis, excluding the terminal leaflets, following standard oil palm morphological measurement procedures. Petiole width and depth were measured at the point of insertion of the lowest rudimentary leaflet, and petiole cross-section (PCS) was calculated as follows:


$$PCS\left(cm^{2}\right)=Petiole\;width\left(cm\right)\times Petiole\;depth\left(cm\right)$$

In the conventional approach, LAI is determined based on three key variables: frond area (a), number of green fronds (N), and planting density (D). To obtain true leaf area, the estimated frond area is corrected using an empirical coefficient of 0.55. For leaf area estimation, different correction coefficients have been reported for distinct palm age classes (Hardon et al., 1969). However, the use of correction factors ranging from 0.51 to 0.57 may result in overestimation of leaf area, which can subsequently affect the accuracy of derived parameters such as fractional light interception (f), light extinction coefficient (k), and radiation conversion efficiency (e) (Squire, 1986). Accordingly, LAI is calculated as the product of these variables (Hardon et al., 1969).


LAI=a x N x D x 0.55

where LAI is the leaf area index (dimensionless), a represents frond area (m² palm⁻¹), N is the number of green fronds per palm, and D is the planting density (palms ha⁻¹). The coefficient 0.55 is an empirical correction factor.

#### 2.3.2 Plant sampling and nutrient analysis

Foliar and rachis sampling were conducted concurrently with vegetative measurements, and samples were collected from all recording palms. Frond selection for nutrient analysis was based on palm age, following established oil palm sampling procedures (Mutert et al., 1999; Corley and Tinker, 2015). Foliar sampling was performed between 0700 and 1230 h in Peninsular Malaysia. After vegetative measurements, the same eight undamaged leaflets used for morphological assessment were collected. Leaflet and rachis samples from each plot were bulked, placed in clean labelled polyethylene bags, and processed on the same day or stored under refrigeration for processing within a few days (Goh et al., 2003). Rachis samples were taken from the same frond as the leaflets. A 10 cm section was cut from the tapering point toward the frond base. After drying, all samples were ground to a fine powder and stored in labelled polyethylene bags prior to analysis. Dried and ground foliar and rachis samples were analysed for nitrogen (N), phosphorus (P), potassium (K), magnesium (Mg), calcium (Ca), and boron (B). Nitrogen was determined by combustion using an elemental analyser at 900 °C (Matejovic, 1993). Phosphorus was determined colorimetrically as a yellow phosphovanadate complex after dry ashing using an Auto Analyser (Varley, 1966). Potassium, magnesium, and calcium were determined by atomic absorption spectrophotometry (AAS) following dry ashing (SIRIM, 1980). Boron was determined by the azomethine method using a UV spectrophotometer after dry ashing with H_2_SO_4_ (John et al., 1975).

### 2.4 Statistical analysis

Data were first inspected for entry errors, outliers, and biological inconsistencies before analysis. Plot-level means were computed from the 15 palms within each plot, and these means formed the basis of subsequent statistical procedures. Data was analysed using analysis of variance (ANOVA) for RCBD. For datasets showing significant treatment effects in ANOVA, post-hoc mean separation was conducted using Tukey’s Honest Significant Difference (HSD) test. Treatment differences were presented using letter grouping notation, where treatments sharing the same letter were not significantly different at α = 0.05. Results were presented as treatment means with standard deviation (SD), accompanied by significance groupings. All statistical analyses were performed using JMP^®^ version 19.0.5 (JMP Statistical Discovery LLC, Cary, NC, USA).

### 2.5 Cost of seedling

Establishment of seedling cost was calculated as the total operational expenditure associated with establishing seedlings in the field during the planting year. This included costs for planting operations, routine field upkeep throughout the establishment year, fertiliser application rounds conducted according to standard agronomic practice, and all labour and material inputs required to implement each treatment.

## 3 Results

### 3.1 Baseline soil properties

The baseline soil was strongly acidic across both depths (pH ~4.4–4.5) with low organic carbon, total nitrogen, exchangeable bases, and CEC, indicating generally poor fertility, while total P was relatively high but accompanied by high variability in available P, suggesting limited and inconsistent phosphorus availability prior to treatment. (**Table 1**).

### 3.2 Response 6 months after planting

#### 3.2.1 Vegetative measurements

No significant treatment effects were observed for total frond number (TF), leaf area index (LAI), or petiole cross-section (PCS). However, rachis length (RL) was significantly influenced by treatment, ranging from 1.25 to 1.38 m. Treatments 1 and 6 recorded the highest RL values (1.38 m), which were significantly higher than Treatment 8 (1.25 m), whereas Treatments 2, 3, 4, 5, and 7 were intermediate and did not differ significantly from either group (**Table 3**).

**Table 3:** Effect of different fertiliser rate and regimes on the total frond, rachis length, leaf area index and petiole cross section at 6 MAP.

Treatment	TF (n)	RL (m)	LAI	PCS (cm^2^)
1	15.30 ± 1.07	1.38 ± 1.07 ^a^	0.15 ± 0.03	2.65 ± 0.28
2	15.38 ± 1.41	1.32 ± 1.41 ^ab^	0.15 ± 0.02	2.59 ± 0.22
3	14.99 ± 1.54	1.37 ± 1.54 ^ab^	0.14 ± 0.01	2.53 ± 0.17
4	14.80 ± 0.89	1.36 ± 0.89 ^ab^	0.15 ± 0.02	2.86 ± 0.47
5	14.72 ± 1.36	1.36 ± 1.36 ^ab^	0.15 ± 0.02	2.46 ± 0.27
6	15.46 ± 1.68	1.38 ± 1.68 ^a^	0.16 ± 0.02	2.77 ± 0.33
7	15.98 ± 2.26	1.35 ± 2.26 ^ab^	0.17 ± 0.03	2.86 ± 0.19
8	15.14 ± 0.73	1.25 ± 0.73 ^b^	0.14 ± 0.01	2.57 ± 0.19

1 Mean values with the same letter within a column are not significant different at p<0.05 by Tukey Test.

#### 3.2.2 Nutrient concentration

At 6 MAP, treatment significantly affected foliar N, P, K, and Mg concentrations, but had no significant effect on foliar B or rachis K (rK). Foliar N concentration was highest in Treatment 8 (3.32%) and lowest in Treatment 7 (2.79%). Similarly, foliar P was highest in Treatment 8 (0.19%), while Treatments 3 and 7 recorded the lowest values (0.17%). Foliar K concentration was also greatest in Treatment 8 (1.51%), whereas Treatments 1, 2, 3, 4, and 6 showed significantly lower values. For foliar Mg, Treatment 6 recorded the highest concentration (0.31%), while Treatment 8 had the lowest value (0.27%). No significant treatment effects were detected for foliar B or rK (**Table 4**).

**Table 4:** Effect of different fertiliser rate and regimes on the N, P, K, Mg, B, rK at 6 MAP.

Treatment	N(%)	P(%)	K(%)	Mg(%)	B(%)	rK(%)
1	3.04 ± 0.13^b^	0.18 ± 0.02^ab^	1.29 ± 0.14^b^	0.28 ± 0.03^ab^	10.29 ± 1.38	1.74 ± 0.25
2	2.97 ± 0.12^bc^	0.18 ± 0.01^ab^	1.32 ± 0.09^b^	0.30 ± 0.02^ab^	10.86 ± 1.57	1.38 ± 0.44
3	3.07 ± 0.20^b^	0.17 ± 0.02^b^	1.30 ± 0.16^b^	0.28 ± 0.03^ab^	12.33 ± 2.94	1.68 ± 0.15
4	3.13 ± 0.12^ab^	0.18 ± 0.01^ab^	1.33 ± 0.11^b^	0.29 ± 0.03^ab^	11.29 ± 1.50	1.54 ± 0.22
5	3.07 ± 0.17^b^	0.18 ± 0.01^ab^	1.38 ± 0.13^ab^	0.28 ± 0.03^ab^	10.86 ± 0.69	1.73 ± 0.25
6	3.16 ± 0.05^ab^	0.18 ± 0.02^ab^	1.36 ± 0.12^b^	0.31 ± 0.03^a^	12.71 ± 1.60	1.40 ± 0.26
7	2.79 ± 0.16^c^	0.17 ± 0.02^b^	1.39 ± 0.18^ab^	0.28 ± 0.02^ab^	10.71 ± 1.38	1.79 ± 0.27
8	3.32 ± 0.10^a^	0.19 ± 0.02^a^	1.51 ± 0.14^a^	0.27 ± 0.03^b^	11.57 ± 1.90	1.56 ± 0.30

1 Mean values with the same letter within a column are not significant different at p<0.05 by Tukey Test.

### 3.3 Response at 12 month after planting

#### 3.3.1 Vegetative measurement

At 12 MAP, treatment had no significant effect on total frond number (TF), leaf area index (LAI), or petiole cross-section (PCS). In contrast, rachis length (RL) was significantly influenced by treatment, ranging from 1.46 to 1.59 m. The highest RL was recorded in Treatment 6 (1.59 m), followed closely by Treatment 7 (1.59 m), whereas Treatment 3 showed the lowest value (1.46 m). Treatments 1, 4, 5, and 8 were intermediate and statistically comparable with both groups, while Treatment 2 was significantly lower than Treatment 6 (**Table 5**).

**Table 5:** Effect of different fertiliser rate and regimes on the total frond, rachis length, leaf area index and petiole cross section at 12 MAP.

Treatment	TF (n)	RL (m)	LAI	PCS (cm^2^)
1	17.80 ± 3.05	1.53 ± 0.09 ^abc^	0.29 ± 0.07	5.57 ± 1.03
2	16.93 ± 2.15	1.47 ± 0.13^bc^	0.25 ± 0.06	4.72 ± 0.84
3	16.22 ± 3.08	1.46 ± 0.11^c^	0.25 ± 0.07	4.76 ± 1.06
4	17.24 ± 1.46	1.54 ± 0.09^abc^	0.27 ± 0.05	5.14 ± 0.89
5	17.02 ± 2.06	1.53 ± 0.06^abc^	0.28 ± 0.05	5.63 ± 1.04
6	17.83 ± 1.47	1.59 ± 0.10^a^	0.30 ± 0.06	5.61 ± 1.05
7	17.77 ± 1.53	1.59 ± 0.04^ab^	0.32 ± 0.05	6.13 ± 1.34
8	16.38 ± 1.81	1.52 ± 0.07^abc^	0.25 ± 0.03	5.14 ± 1.05

1 Mean values with the same letter within a column are not significant different at p<0.05 by Tukey Test.

#### 3.3.2 Nutrient concentration

At 12 MAP, treatment significantly affected foliar N, P, and K concentrations, but had no significant effect on foliar Mg, B, or rachis K (rK) (**Table 6**). The highest foliar N concentration was recorded in Treatment 7 (3.02%), followed by Treatment 8 (2.82%), whereas Treatments 1 to 6 were significantly lower. Foliar P showed a similar pattern, with Treatments 7 and 8 recording the highest concentrations (0.17%), while Treatments 1, 2, 3, and 4 were among the lowest. For foliar K, Treatment 7 produced the highest concentration (1.54%), followed by Treatment 8 (1.44%), whereas Treatments 1 and 3 recorded the lowest values (1.20%). No significant differences among treatments were observed for foliar Mg, B, or rK.

### 3.4 Cost analysis and labour productivity

Total field establishment cost per seedling differed among treatments, ranging from RM 9.58 in T1 to RM 19.36 in T8 (**Table 6**). Costs were relatively similar between T2 and T3, and between T4 and T5, while T6, T7, and T8 showed the highest establishment costs (**Table 7**). These differences reflected variation in treatment-related field operations and material inputs during planting and early establishment.

**Table 6:** Effect of different fertiliser rate and regimes on the N, P, K, Mg, B, rK at 12 MAP.

Treatment	N(%)	P(%)	K(%)	Mg(%)	B(%)	rK(%)
1	2.37 ± 0.20^b^	0.14 ± 0.02^bc^	1.20 ± 0.15^c^	0.30 ± 0.04	6.86 ± 0.90	1.26 ± 0.30
2	2.39 ± 0.14^b^	0.14 ± 0.00^bc^	1.24 ± 0.13^bc^	0.31 ± 0.04	7.57 ± 1.13	1.26 ± 0.23
3	2.25 ± 0.23^b^	0.14 ± 0.01^bc^	1.20 ± 0.15^c^	0.31 ± 0.05	7.00 ± 0.89	1.25 ± 0.15
4	2.52 ± 0.13^b^	0.15 ± 0.01^bc^	1.26 ± 0.11^bc^	0.30 ± 0.03	7.86 ± 0.90	1.22 ± 0.20
5	2.51 ± 0.19^b^	0.15 ± 0.01^abc^	1.26 ± 0.06^bc^	0.31 ± 0.03	8.00 ± 0.82	1.32 ± 0.24
6	2.50 ± 0.09^b^	0.15 ± 0.01^abc^	1.27 ± 0.04^bc^	0.31 ± 0.04	7.86 ± 1.07	1.22 ± 0.23
7	3.02 ± 0.12^a^	0.17 ± 0.01^a^	1.54 ± 0.15^a^	0.31 ± 0.04	7.86 ± 0.69	1.52 ± 0.34
8	2.82 ± 0.24^a^	0.17 ± 0.01^a^	1.44 ± 0.15^ab^	0.33 ± 0.04	7.71 ± 1.25	1.56 ± 0.23

1 Mean values with the same letter within a column are not significant different at p<0.05 by Tukey Test.

**Table 7:** Fertiliser related establishment cost (RM seedling^-1^) during the planting year inclusive of fertiliser materials and application labour.

Fertiliser	Cost/seedling (RM)
T2	9.58
T4	11.30
T1	11.36
T3	13.79
T6	13.88
T7	17.08
T5	17.43
T8	19.36

1 T1, T3 & T5-(CRF A); T2, T4 &T6-(CRF B); T7-Conventional fertiliser 4 round application; T8-Conventional fertiliser + Mulching.

## 4 Discussion

### 4.1 Response of immature oil palm to fertiliser strategy

This study evaluated fertiliser strategy effects on immature oil palm under hilly terrain conditions, where treatment responses were assessed through vegetative growth traits and foliar nutrient concentrations rather than yield. This approach is agronomically justified given that immature palms prioritise canopy establishment and root system development over reproductive output during the early post-planting phase (Peng et al., 2022). Vegetative and nutritional benchmarks therefore serve as the most relevant proxies for early establishment performance, as they reflect the nutritional foundation upon which subsequent productive capacity is built (Mohidin et al., 2015; Kamireddy et al., 2023).

Across treatments, responses were more consistently expressed in foliar nutrient concentrations than in vegetative traits. Among the growth parameters, only rachis length showed a significant and consistent treatment responses, while total frond number, leaf area index, and petiole cross-section remained largely undifferentiated across treatment. This pattern suggests that, within the first year after planting, differences in nutrient availability are more sensitively reflected in plant tissue composition than in broader structural development. This interpretation is consistent with the established use of foliar analysis as a sensitive and early diagnostic indicator of nutrient status in oil palm, while canopy-scale parameters such as LAI and rachis length capture the cumulative expression of structural expansion over time, which may require longer monitoring horizons to detect treatment-driven differentiation (Mutert et al., 1999; Fairhurst and Härdter, 2003b; Tarmizi and Mohd Tayeb, 2006; Dubos et al., 2019).

### 4.2 Early nutrient and growth responses at 6 MAP

At 6 MAP, treatment effects were more clearly expressed in foliar nutrient concentrations than in vegetative measurements, with significant differences recorded for foliar N, P, K, and Mg. Among vegetative parameters, only rachis length showed a significant treatment response at this stage.

The higher foliar N, P, and K concentrations observed under the conventional fertiliser programme applied on the soil surface with mulch cover (T8) suggest that the combination of localised placement and surface organic cover enhanced nutrient retention and availability within the near-surface rooting zone. During the early post-planting phase, the developing root system of immature oil palm is predominantly concentrated in the upper soil layers proximal to the planting hole. The mulch layer likely reduced the velocity and volume of surface runoff, thereby limiting fertiliser displacement and promoting *in situ* mineralisation and nutrient release near the active root zone (Formaglio et al., 2021; Harahap et al., 2025). This interpretation is further reinforced by the contrasting nutritional outcome observed under T7, which received the same conventional fertiliser source through four split broadcast applications on bare soil surface. The lower foliar NPK concentrations under T7 compared to T8 at 6 MAP indicate that surface cover may have influenced nutrient retention and early plant-available nutrient pools, even when fertiliser source and total rate were held same.

In contrast, the highest foliar Mg concentration and greater rachis length recorded under the high rate CRF applied in the planting hole T6. Despite receiving a lower overall fertiliser volume compared with T8, T6 demonstrated better Mg nutrition and early vegetative development. This suggests that the controlled-release characteristics of CRF, combined with subsurface placement proximal to the developing root system, likely enhanced nutrient interception efficiency and sustained Mg availability when root exploration was still limited (Timilsena et al., 2015; Chen and Wei, 2018).

### 4.3 Nutrient and growth responses at 12 MAP

By 12 MAP, the overall pattern of treatment responses continued to be predominantly expressed in foliar nutrient status rather than in canopy-scale vegetative traits. Total frond number, LAI and petiole cross-section did not differ among treatments, whereas rachis length (RL) showed a modest but significant response (1.46–1.59 m), suggesting that early structural differentiation was expressed mainly through frond elongation rather than changes in frond production or canopy density (Rasid et al., 2014; Sugianto et al., 2023).

Foliar N, P, and K showed clear treatment separation at 12 MAP, with conventional fertiliser treatments (T7 and T8) recording the highest concentrations, while all CRF treatments (T1–T6) remained at lower levels. This consistent pattern indicates that, across the range of CRF formulations and rates tested, the CRF treatments did not match the foliar NPK status achieved by conventional fertiliser regimes at this assessment point. This finding is important for interpretation that the comparable vegetative performance observed in selected CRF treatments relative to conventional programmes must be evaluated alongside this nutritional context and should not be interpreted as evidence of nutritional equivalence.

The lower foliar NPK concentrations under CRF treatments may reflect the differences in nutrient release and replenishment dynamics between CRF and conventional fertiliser strategies. CRF was applied as a single basal application at planting, with the expectation that polymer-coated or resin-coated granules would release nutrients progressively in accordance with soil temperature and moisture conditions. In contrast, conventional treatments were either replenished through repeated multiple split applications throughout the year (T7) or maintained near-surface nutrient pools through the mulch-retention mechanism (T8), both of which would have sustained greater short-term nutrient availability proximal to the assessment period (Formaglio et al., 2021; Harahap et al., 2025).

In contrast to NPK, foliar Mg, B, and rachis K concentrations did not differ significantly among treatments at 12 MAP. This nutrient-specific pattern indicates that treatment effects at 12 MAP were concentrated in the macronutrient NPK fraction, while Mg, B, and tissue K in rachis tissue were maintained within a sufficiency range across all programmes.

Notably, RL was highest under T6 (comparable to T7) despite lower foliar NPK than T7, suggesting that rachis elongation may reflect integrated nutrient supply and early root and fertiliser contact over time rather than instantaneous foliar nutrient concentration alone, particularly under localised subsurface placement strategies (Timilsena et al., 2015; Chen and Wei, 2018). Under T6, the high-rate CRF placed directly in the planting hole would have provided sustained nutrient release in close proximity to the developing root system throughout the early establishment phase, potentially supporting rachis elongation through a more consistent supply of nutrients during the critical period of frond initiation and extension, even if instantaneous tissue concentrations had declined by 12 MAP. This interpretation highlights a fundamental limitation of single time-point foliar sampling in capturing the full nutritional history relevant to structural growth and underscores the need for repeated foliar assessments at multiple developmental stages to adequately characterise the nutrient-growth relationship under contrasting fertiliser strategies.

### 4.4 Agronomic implications for fertiliser management strategy

The collective 12 MAP findings indicate that fertiliser management exerted stronger effects on nutritional status than on whole-canopy morphology within the first year after planting. This pattern is consistent with the view that vegetative growth in oil palm is relatively buffered against moderate nutritional variation during early establishment, and that longer monitoring periods (Dubos et al., 2019; Arifin et al., 2022). Since treatment differences were more consistently expressed in foliar nutrient concentrations than in morphological traits, strategies that maintained superior tissue nutrient status even in the absence of proportionally greater structural growth should be regarded as agronomically meaningful, as they reflect a nutritional foundation with implications for longer-term palm development and productive performance (Koussihouèdé et al., 2020; Peng et al., 2022).

Selected CRF treatments supported vegetative establishment outcomes comparable to the conventional programme within the first year, although conventional treatments maintained higher foliar NPK concentrations at 12 MAP (Bah, 2015). The equivalent structural establishment under lower foliar NPK concentrations in CRF treatments raises the question of whether CRF-supported palms were operating at a nutritionally suboptimal but functionally adequate level during this phase, or whether the lower concentrations reflect a different pattern of nutrient allocation and utilisation that nonetheless fulfilled structural growth requirements. Although CRF is broadly associated with improved synchronisation between nutrient release and crop demand, which would theoretically reduce luxury consumption and concentrate nutrient uptake near periods of peak demand, nutrient use efficiency (NUE) was not directly quantified in this study through isotopic or mass balance methods. The present findings should therefore not be presented as evidence of improved NUE. Rather, they indicate that selected CRF treatments were capable of maintaining early vegetative establishment within the first year with lower fertiliser input (Bah, 2015; Pratiwi et al., 2026).

The absence of proportional vegetative response to increasing CRF rates also warrants careful interpretation. Where intermediate or lower CRF rates achieved nutrient status and growth outcomes comparable to higher rates, this suggests that nutrient supply had reached, or approached, the threshold adequate for early establishment requirements, and that further rate increases did not yield commensurate agronomic benefit within the first year (Purwanto et al., 2018; Satriawan et al., 2019). This is agronomically significant, as it implies that fertiliser rate optimisation targeting sufficiency rather than excess is both economically prudent and environmentally appropriate, particularly given the elevated loss risk on sloped terrain (Lim et al., 2023; Harikumaran and Vijayalakshmi, 2025).

Given that oil palm begins producing fruit bunches only approximately three years after field establishment, yield data were unavailable at this stage, and cost-benefit analysis could therefore only be interpreted through the calculation of first-year input establishment costs. The first-year establishment input cost analysis revealed a substantial range across treatments, from RM 9.58 seedling⁻¹ (T2) to RM 19.36 seedling⁻¹ (T8). This cost gradient indicates that higher-input programmes are only economically defensible when they deliver measurable agronomic gains within the evaluation period. Within the CRF programme, treatments T1 to T6 ranged from RM 9.58 to RM 17.43 seedling⁻¹, compared with RM 17.08 seedling⁻¹ for the conventional four-round programme T7 and RM 19.36 seedling⁻¹ for the mulching plus fertiliser treatment T8. Importantly, the cost advantage of CRF was formulation dependent: CRF B (T2, T4, T6) was consistently lower-cost (mean RM 11.59 seedling⁻¹), whereas CRF A (T1, T3, T5) showed higher and more variable costs (mean RM 14.19 seedling⁻¹) and included one treatment (T5) marginally higher than the conventional programme.

Beyond direct cost considerations, CRF offers a distinct operational advantage in hilly plantation environments through the reduction in the frequency of fertiliser application field operations. In conventional programmes, split applications require multiple entries into steep terrain, which is a logistically demanding, labour-intensive, and time-consuming requirement that is compounded by the limited accessibility. CRF, applied as a single basal treatment at planting, substantially reduces these operational demands while simultaneously providing nutrients over an extended period. Furthermore, the localised and controlled nutrient release characteristics of CRF reduce the temporal overlap between high nutrient availability and high runoff events, potentially improving nutrient retention efficiency at the plot scale relative to soluble broadcast applications on sloped land. These combined operational, economic, and environmental considerations position CRF, particularly lower-cost formulations such as CRF B as a practically relevant and agronomically defensible fertiliser management option for immature oil palm establishment on hilly terrain.

## 5 Conclusion

This study demonstrated that selected CRF treatments supported vegetative establishment of immature oil palm on hilly terrain to a level broadly comparable with conventional fertiliser programmes within the first year after planting, with treatment effects expressed more consistently in foliar nutrient concentrations than in canopy-scale morphological traits. While conventional programmes maintained higher foliar NPK concentrations at 12 MAP, this nutritional difference did not translate into proportional deficits in the vegetative growth parameters assessed, suggesting that CRF-supplied nutrients (as low as 500 g of CRF per palm) were adequate to meet early structural establishment requirements.

## 6 Suggestion for future research

Future research should prioritize the performance validation of controlled release fertilisers (CRF) across diverse locations encompassing a range of soil types and climatic conditions, to better characterize variability in crop responses. In addition, extended establishment periods should be evaluated to determine whether differences in nutrient uptake and vegetative growth translate into early yield advantages. Further investigation into the release mechanisms of various CRF formulations is also warranted. Field-based studies are needed to quantify how environmental factors, particularly temperature and soil moisture, influence nutrient release patterns and subsequent plant uptake. Finally, future work should incorporate comprehensive environmental assessments, including the quantification of nutrient loss pathways such as leaching and runoff. This will help determine whether improvements in nutrient use efficiency are consistently associated with reductions in nutrient losses under commercial field conditions.

## Data Availability

The data are not publicly available due to estate confidentiality but are available on reasonable request. Requests to access the datasets should be directed to bernard.khoo@sdguthrie.com.
